# Special Considerations for the Treatment of Pain from Torture and War

**DOI:** 10.1007/s40140-016-0187-0

**Published:** 2016-10-25

**Authors:** Amanda C. de C Williams, Emma Baird

**Affiliations:** 1 Research Department of Clinical, Educational and Health Psychology, University College London, Gower Street, London, WC1E 6BT UK; 2 Lancashire Teaching Hospitals NHS Foundations Trust, Sharoe Green Lane North, Fulwood, Preston, Lancashire PR2 9HT UK

**Keywords:** Military veterans, Refugees, Trauma, Post-traumatic stress, PTSD, Battlefield, Acute pain, Chronic pain, Persistent pain, Injury, Rehabilitation

## Abstract

Pain care for survivors of torture and of war shows similarities and marked differences. For both, pain can be complex with unfamiliar presentations and the pains hard to assign to known disorders. For many survivors, pain and associated disability are overshadowed by psychological distress, often by post-traumatic stress symptoms that can be frightening and isolating. Pain medicine in war can exemplify best techniques and organisation, reducing suffering, but many military veterans have persistent pain that undermines their readjustment. By contrast, survivors of torture rarely have any acute health care; their risk for developing chronic pain is high. Even when settled as refugees in a well-resourced country, their access to healthcare may be restricted. Recent evidence is reviewed that informs assessment and treatment of pain in both groups, with the broader context of psychological distress addressed at the end. Clinical and research implications are briefly outlined.

## Introduction

Although there are many differences in how we understand and seek to treat pain from torture and from war injury, the shared background of psychological trauma is highly relevant to how the patient is best helped with pain, and what treatment he or she is likely to attempt. Understanding of psychological trauma is addressed in a section that follows the separate considerations for treatment of pain from torture and from war. Patients with pain from either are likely to present in mainstream health services, but providing the best care requires considerations beyond everyday practice. While assessment and treatment broadly follow the same principles as for any patient, understanding of particular aspects of injury, and of early treatment in the case of war injuries, is helpful to the clinician, and the clinician’s appreciation of the wider context of the injury experiences will help the patient to feel understood and to engage with treatment and rehabilitation attempts.

## Pain from Torture

Torture and organised violence are offences against human rights, rights that we all have by virtue of being human, regardless of our civil status or other characteristics. Two fundamental freedoms—not to be tortured and not to be enslaved—are absolute and cannot be derogated under any circumstances, unlike other rights, such as to life, liberty, or health care. However, enforcement of these rights and freedoms is a different matter. The UN Convention on Torture (UNCAT) [1] is only binding to those countries that sign up to it, although countries that currently use torture include both signatories and non-signatories [2, 3].

We know remarkably little about the physical effects of torture, including persistent pain; there has been far more study of psychological effects, albeit often narrowly defined, and of interventions for these. Although some torture is inflicted in public settings, without the fear of restorative justice, or is redefined as “not torture” by the responsible authority, as in the case of waterboarding (effectively drowning) by US agencies, much torture takes place away from any scrutiny and is not recorded. The survivors (and most people do not survive torture) often find it hard, cognitively or emotionally, to recall and to describe what happened to them. This means that we often struggle to understand what was done to the survivor and, even if we understand what was done, to match it to any known model in human or other animal research. In this sense, we have to start from first principles with the patient who has been tortured and now has pain, and build as coherent a model as we can of pain mechanisms, recent or lasting damage, and the individual’s psychological and social state, in order to arrive at proposals for treatment.

The first task is to recognize the torture survivor: we know that most go unrecognized [4, 5]. It is often estimated that at least 30 % of refugees have been subject to torture or organized violence, yet refugees are rarely asked about experiencing violence. It is very unlikely that a refugee who has not been subjected to violence would be offended by being asked, while for the refugee who has experienced violence, the question indicates that the clinician is aware and able to listen should he or she wish to disclose at a subsequent encounter. The question should ask clearly about whether the patient was badly treated either in country of origin or in flight and is ideally backed by information about the patient’s country of origin (such as is easily available on the internet). Many clinicians report hesitating to ask, sometimes from fear of causing offence, but more often from fear of disclosure for which they feel practically (in a brief consultation), and often emotionally, unprepared. Yet the duty to provide best possible health care cannot be put aside because of these difficulties. Further, asylum seekers suffer inequities in access to health care [6], and there is little research on conditions that can mitigate these inequities.

World Medical Association (2006) defines torture as “the deliberate, systematic or wanton infliction of physical or mental suffering by one or more persons acting alone or on the orders of any authority, to force another person to yield information, to make a confession, or for any other reason” [7].

### Assessing Pain

Assessment for asylum claims is a specialized area not addressed here: the interested reader is directed to literature on medicolegal reports, particularly *The Manual on the Effective Investigation and Documentation of Torture and other Cruel, Inhuman or Degrading Treatment or Punishment* (The Istanbul Protocol), drafted in 1999 [8]. Medicolegal assessment requires that problems identified by the examining clinician can be associated with certainty with the torture reported, a deeply problematic criterion for pain and other symptoms. Outside this setting, the clinician should adjudicate with caution on the correspondence of the patient’s current complaints with his or her history of ill-treatment.

Estimates of persistent pain from torture are high, several over 80 %, and these in a population whose mean age is lower than that of most chronic pain patients and who are predominantly male [9, 10]. Headache and musculoskeletal pain are the two most commonly reported, including widespread musculoskeletal pain [11], foot pain [11, 12] and pelvic pain [10] likely to be related to specific as well as general methods of torture.

Assuming that the patient can describe his or her torture, it is still far from straightforward to make sense of the pain. The possibility of lasting structural problems, such as damaged joints and malunited fractures, or of infections or other disease should be considered. The victim of torture is usually held in very poor conditions, with inadequate nutrition, extremes of temperature, poor hygiene and pervasive stress. Assessment needs to be thorough and may take several sessions, limited not only by time but also by how much the patient can handle. It should also be borne in mind that loss of consciousness is common during torture, or the patient may have sustained brain damage affecting recall and other functions.

Other than the careful account by Rasmussen [13, 14] of the medical problems of 200 torture survivors, the most useful studies of pain from torture are those of foot pain from falanga (beating the soles of the feet) and shoulder and upper limb pain from suspension by the arms (see overview [15]). These studies consider the immediate effects of physical trauma, the longer term type of pain, the mechanisms in soft tissue, connective tissue and nervous tissue likely to contribute, and secondary effects. For example, falanga often leaves a neuropathic pain (with signs on quantitative sensory testing: [16]) in the sole of the foot that may worsen with weight bearing or may be more problematic when not bearing weight, such as in bed, a deep pain that extends up the leg when walking and distorted gait to avoid contact of the sole of the foot with the ground that can then affect knee and hip [17–19]. Suspension by the arms, often extended behind the body, can produce abnormal sensory symptoms such as a feeling of heaviness of the arm, and avoidance of use, as well as pain and instability in the shoulder that is most probably a combination of overload of the joint tissues with partial lesion of the brachial plexus [20].

Such attempts to make sense of pain mechanisms are valuable to other clinicians, even from a small sample. Neuropathic pain may be particularly common in this population [20, 21•, 22–24], but is peculiarly subject to being dismissed as psychosomatic by those with poor understanding of pain and of risk factors for pain, including extreme stress, sleep deprivation and traumatic head injury [25]. Though headache is one of the commonest complaints from torture survivors, there is a lack of studies [11] and Sharp and Jenkins [26] cautioned against dismissing the symptom as self-limiting “post-concussion syndrome”. They reproduce helpful guidelines for investigation of mild traumatic brain injury, to which headaches are often attributed by the survivor, as well as criteria for classifying traumatic brain injury as moderate or severe. Likewise, it is important to ask both women and men about sexual violence and rape, often used strategically to undermine survivors’ family and marital relationships [27] and a commonly attributed cause of pelvic pain in survivors [10].

Assessment of the patient’s pains, the history of the pains and the patient’s beliefs about those pains may reveal issues where the individual can, on the basis of evidence, be reassured about fears of continuing or unhealed damage. It is unsurprizing that fears of unhealed damage are relatively common in physically traumatized individuals.

### Treating Pain

There is a dearth of published research on treating pain from torture. A systematic review [28•] found only three treatments for chronic pain in torture survivors, two of which consisted of cognitive behavioural therapy with biofeedback [29, 30], but with no benefits for pain, disability, and distress after treatment, and only very weak effects on pain at follow-up [29]. The third study was of hands-on physiotherapy [31] with no reduction in pain despite reported improvements in disability and distress, but this was a small and likely underpowered study. Non-randomized trials included several of pain school (education on pain management, with or without exercise instruction, e.g. [32]), some culturally adapted, but largely with modest or no improvements. Many studies gave the impression of having set out to test a particular treatment, regardless of the presenting problems of the target population or of the particular patients recruited. Nevertheless, physical therapies may be more acceptable to patients than psychologically based interventions, both on the basis of cultural familiarity (e.g. [33]) or of being unthreatening and even soothing [34].

### Summary

Since the systematic review and other recent narrative reviews [35, 36] offer no guidance on treatment, nor have any randomized studies been found for pharmacotherapy or other mainstream treatments for chronic pain from torture, there is no reliable evidence to guide treatment. The clinician should therefore proceed as with any other patient, assessing the pain, trying to identify treatment mechanisms, and applying treatment for which there is good evidence. It would be helpful if the clinician could assemble carefully recorded casework and submit it to a central source so that over time outcomes could be analysed and compared. Such findings can then become the basis for better targeted prospective studies.

## Pain from War

Over recent decades, there have been significant improvements in the management of trauma occurring in armed conflict. The number of combat zone deaths has reduced from 24 % of casualties in the US-Viet Nam war to around 10 % in the recent conflicts in Iraq and Afghanistan [37]. With these improvements, there has been an increase in the numbers surviving with more extensive injuries. Among US troops, the State Department recorded 20,083 wounded in Afghanistan alone [38]. The need to provide these patients with adequate pain relief from the time of injury has taken increasing priority: effective analgesia in the field is not solely for humanitarian reasons, although they are important, but patients who are comfortable are easier to move and make less noise, potentially of critical importance during covert extraction. The link between acute pain and chronic or persistent pain is well-established, and, although the mechanisms are not fully understood, adequate treatment of acute pain reduces the likelihood of development of persistent pain [39]. Good analgesia in the acute phase is also associated with a reduction in secondary complications (DVT/PE, pneumonia, MI) by improving the patient’s ability to mobilize, to take deep breaths and cough, and by reducing the body’s stress response to the injury [40]. It will also aid the rehabilitation process.

### Battlefield Analgesia

Morphine was first marketed in 1827 and has been used in multiple combat situations since to treat pain. All British soldiers are provided with and taught how to use a morphine auto-inject, a spring-loaded syringe that delivers 10 mg of morphine (in 0.7 ml) IM. It remains one of the first line analgesic drugs used by the US military in the field. Depending on the skills available in the field, further morphine may be given along with paracetamol, NSAIDs and ketamine. Recently, the UK military have introduced 400 μg fentanyl lozenges as an alternative to morphine, the latter being associated with complications that may lead to significant morbidity and mortality in the battlefield and during the immediate transfer of a wounded patient. In these environments, adequate personnel for monitoring may not be available, leaving respiratory depression and sedative effects of opioids unrecognised. Opioids are also known to cause immunosuppression and may lead to susceptibility to Acinetobacter infection [41], a major cause of wound infection in this population. For all these reasons, modern conflict analgesia is steering away from opioids and towards multimodal analgesia and novel ways to treat pain.

### Base Hospital Treatment

Regional anaesthesia is increasingly playing a major role in the treatment of pain in a conflict setting. A variety of techniques is used: central neuraxial blocks, continuous peripheral nerve block catheters and single injection nerve blocks. In the war zone, placement of these blocks has become easier with the advent of portable ultrasound guidance technology. Regional anaesthesia is thought to offer superior analgesia to parenteral routes when carried out appropriately, reducing the risk of respiratory depression and sedation. Novel pumps to deliver local anaesthetic via a peripheral nerve catheter have been specifically designed for military use; the pumps are programmable, lightweight, can operate at high altitude, and do not interfere with other military equipment. The main risks associated with regional anaesthesia in this context are masking of compartment syndrome, coagulopathy and infection. For patients thought to be susceptible to compartment syndrome, surgical teams carry out elective prophylactic fasciotomies to mitigate the risk. Patients who have undergone neuraxial blockade are assessed according to specific guidelines to ensure that an epidural hematoma or abscess is not missed. Monitoring is also done for local anaesthetic toxicity. In the US military, any catheter that has been placed in the field is removed within 3 days of arriving at the medical centre to minimize infection risk. There is adherence to US and UK national guidelines on the placing of regional anaesthetic blocks and catheters in patients on anticoagulation therapy.

### Further Treatment Options

Ketamine is an *N*-methyl-d-aspartate (NMDA) receptor antagonist, a powerful analgesic and anaesthetic agent that has proved useful in trauma patients due to its relative cardiovascular stability with preservation of laryngeal reflexes and relatively little effect on respiratory drive. It can be given orally, intramuscularly, intranasally and intravenously (as a bolus or as an infusion). It has been postulated to prevent the progression of acute to chronic pain [42] but this requires further research. The main risk with ketamine is of hallucinations, a relevant problem for patients who have suffered psychological trauma and who may still be in a stressful environment.

Multimodal pain management is expected in war environments as in civilian medicine. NSAIDs can reduce opioid requirements along with paracetamol (acetaminophen), and ketorolac as an intranasal preparation [43] may be an additional resource. Gabapentin and pregabalin, widely used to treat acute and chronic neuropathic pain, are also used in some surgical specialties with the aim of pre-empting progression from acute pain to persistent pain, but their role in trauma patients is so far unassessed.

Short-acting opioids delivered in novel ways with potential in the battlefield setting are fentanyl via a patient-controlled transdermal device [44] and sublingual administration of sufentanil [45]; both delivery systems avoid the need for intravenous access and reduce the risk of administration errors. Phase three clinical trials are currently underway for a new formulation of bupivacaine liposome [46], to establish whether this formulation lasts significantly longer than the standard drug. If single shot local anaesthetic blocks could be made to last longer, the need for catheter placement with the associated risks could be avoided. Finally, some individuals in the US and UK military have developed the concept of battlefield acupuncture, placing acupuncture needles in the patient’s auricular region to alleviate pain, but evidence for its usefulness on the battlefield is anecdotal at present.

Upon arriving back in the UK or USA, each patient’s acute pain is managed by a multidisciplinary pain team with the aim of optimal pain relief, prevention of transition to persistent pain and rehabilitation. For patients who do develop persistent pain, treatment follows national guidelines and referrals are made to a chronic pain service.

### Summary

A strategy for pain management that commences on the battlefield and continues throughout the patient’s evacuation and repatriation is now the gold standard of care for the modern military [47••]. Clearly more humane, this also aids the safe transfer of wounded individuals, helps prevent the progression of acute pain to chronic pain and facilitates their rehabilitation. It provides an exemplar for hospital treatment of trauma in the UK [48], and treatment of major trauma has already been shaped by lessons learned on the battlefields of Iraq and Afghanistan. This is just one more example of how clinical practice in areas of peace can be improved by learning from areas of conflict [49••, 50••].

However, not all medicine in war is so well-resourced. Surgeons who stayed in Sarajevo during the siege of 1992–1995 had no previous experience of war injuries but had to treat injuries from sniper fire, grenades and other explosions. Much surgical work was done, with the help of anaesthetists, under regional rather than general anaesthesia because of the poor condition of many patients. When anaesthetic supplies ran out, surgery was done with no anaesthesia. Nevertheless, during that time they initiated a large-scale rehabilitation programme with contributions not only from medical personnel but from physiotherapists and psychologists, aiming at multidisciplinary treatment, often for phantom pain [51, 52].

## Psychological State

The 1914–18 war was the beginning of post-traumatic formulations, initially as “shell shock” although not uniquely associated with exposure to explosions. Part of the legacy from that era is the association with moral or psychological weakness of those who experience symptoms. This is profoundly unhelpful to those affected and a deterrent to reporting symptoms, particularly among military personnel, and development after the US–Viet Nam war was also partly driven by political concerns [53]. However, we lack an understanding of why some people exposed to a terrifying event or events suffer these symptoms afterwards while others exposed to the same event at the same time report no symptoms, or their symptoms rapidly and spontaneously remit. The diagnosis is entirely based on symptom report, with no biological markers [54•, 55••, 56].

Torture is usually a repeated experience (if only by the ever-present threat of repetition, or witnessing it being inflicted on others), a terrifying and inescapable threat to life, health and sanity. It spreads distrust, between survivors and between them and their families and communities, undermining resources that might otherwise support recovery. Military veterans, by contrast, appear to present greater problems with alcohol misuse [57], risky behaviours and threatened or actual violence to those around them [58], as well as feelings of loss not only for comrades who died but for the comradeship and commitment that characterised military life. There are overall more similarities than differences among UK and US military personnel returning from war [59].

Yet much research on the psychological sequelae of torture subsumes effects under the label post-traumatic stress disorder (PTSD), a hugely heterogeneous category [60], despite the multiple other problems recorded in studies of torture survivors [61] and refugees [62]. Further, intervention studies are dominated by trials that recruited civilian populations that had experienced traumatic, but almost always single, events (such as a serious road traffic accident or assault) in the context of an otherwise stable life, circumstances very different from the tortured detainee or the soldier in a war zone.

Characteristic problems associated with trauma are re-experiencing events as if in the present (flashbacks), when cued by sounds (screams, slamming doors), smells (burning), visual cues (uniforms, blood), and others; avoidance of situations where those cues may be encountered; a high level of vigilance to threat, suspecting threat from ambiguous cues; and nightmares. It is common to encounter more generalized anxiety and distrust, depression and profound lack of motivation, accompanied by difficulty finding meaning in everyday life.

The emphasis on psychological problems has also propagated myths about pain that will be distressingly familiar to the pain clinician: that if there are no physical signs, then there can be no pain except factitious or imagined pain (also referred to as “psychosomatic”, “medically unexplained”, etc.), in defiance of 50 years of pain science that describes changes in the nervous system that perpetuate and amplify pain (e.g. [63]). The consistent risk factors for chronic pain are high intensity pain and high levels of distress, conditions that almost define torture and are widespread in war-related injuries.

While the patient’s psychological state is relevant during assessment and may be even more important in devising a treatment plan, attempts to assign pain to purely psychological causes should be resisted. Explaining pain mechanisms is challenging, even when clinician and patient share a language and culture; without either it can be very difficult and unsatisfactory but should be attempted. We urgently need dynamic and accessible resources to support such explanations.

### Post-Traumatic Stress: Models and Treatments

It is common for both survivors of torture and war trauma with chronic pain and disabling post-traumatic symptoms to find themselves between mental and physical health services, neither of which wishes to offer treatment until the other has effectively treated them. Because symptoms of post-traumatic stress can complicate treatment of chronic pain, and chronic pain can be an obstacle to treating post-traumatic stress symptoms, treatment is ideally integrated (see review by Bosco et al. [64]), even if delivered by different services. Combined treatment within a single service for chronic pain and post-traumatic symptoms has shown benefits for both pain and psychological state (e.g. [65]), but is very rarely available.

What the clinician assessing and treating pain needs to appreciate is that the field of post-traumatic stress is subject to strongly held beliefs, with some vested interests propagating particular treatment models [66], without either evidence for their superiority in general [67] or their suitability for torture survivors and military veterans. A systematic review and meta-analysis [67] found no immediate treatment effects of psychological treatments in nine randomised controlled trials on post-traumatic symptoms, general distress, or quality of life in torture survivors, and only very weak effects on post-traumatic symptoms and distress at follow-up. The trials were mostly of narrative exposure therapy, currently the dominant model and subject to critical commentary [67, 68, 69•].

## Conclusions

Clinically, military medicine has improved hugely in its understanding and treatment of pain, with benefits across the medical field. Yet, pain management in most parts of the world, including many where millions suffer torture and organised violence that creates pain and disability, is poorly resourced and often absent from the public health agenda. Many refugees who have been tortured come from such settings, and clinicians need to be proactive in asking about, and being willing and able to listen to, their experiences. That may require time, interpreters, and other resources in short supply, but which are required in order to provide equitable and accessible health care.

Chronic pain affects both torture survivors and military veterans, yet the extent of their distress, instability and day to day struggles can make it difficult for them to work at pain management methods that offer them a better quality of life. Further, many of our medical colleagues continue to dismiss chronic pain in which there are no physical signs or where there is manifest psychological distress. This is a very serious disservice to the patient and requires that pain specialists advocate for pain management across clinical settings.

In research terms, we need to keep an open mind about ways of understanding and treating post-traumatic symptoms in people with persistent pain, whether from torture or war injuries, while using the best evidence available from related mental health fields. Treatments need to aim not only to relieve pain, by whatever medical means, but also to reduce distress and disability and improve quality of life. We often lack suitable instruments for evaluation that are culturally appropriate and in the language of the patient [69•]. The lack of literature on treatment of pain from torture, and from war, compared to that on treatment of psychological problems, leaves us in a position where even case reports represent an advance in shared knowledge. While that may seem discouraging, it widens the field of those who can contribute to all pain clinicians.
